# Characterisation of the *Trichinella spiralis* Deubiquitinating Enzyme, TsUCH37, an Evolutionarily Conserved Proteasome Interaction Partner

**DOI:** 10.1371/journal.pntd.0001340

**Published:** 2011-10-04

**Authors:** Rhiannon R. White, Sachiko Miyata, Eliseo Papa, Eric Spooner, Kleoniki Gounaris, Murray E. Selkirk, Katerina Artavanis-Tsakonas

**Affiliations:** 1 Division of Cell and Molecular Biology, Department of Life Sciences, Imperial College London, London, United Kingdom; 2 Health Sciences and Technology, Harvard/Massachusetts Institute of Technology (MIT), Cambridge, Massachusetts, United States of America; 3 Whitehead Institute for Biomedical Research, Cambridge, Massachusetts, United States of America; McGill University, Canada

## Abstract

**Background:**

*Trichinella spiralis* is a zoonotic parasitic nematode that causes trichinellosis, a disease that has been identified on all continents except Antarctica. During chronic infection, *T. spiralis* larvae infect skeletal myofibres, severely disrupting their differentiation state.

**Methodology and Results:**

An activity-based probe, HA-Ub-VME, was used to identify deubiquitinating enzyme (DUB) activity in lysate of *T. spiralis* L1 larvae. Results were analysed by immuno-blot and immuno-precipitation, identifying a number of potential DUBs. Immuno-precipitated proteins were subjected to LC/MS/MS, yielding peptides with sequence homology to 5 conserved human DUBs: UCH-L5, UCH-L3, HAUSP, OTU 6B and Ataxin-3. The predicted gene encoding the putative UCH-L5 homologue, TsUCH37, was cloned and recombinant protein was expressed and purified. The deubiquitinating activity of this enzyme was verified by Ub-AMC assay. Co-precipitation of recombinant TsUCH37 showed that the protein associates with putative *T. spiralis* proteasome components, including the yeast Rpn13 homologue ADRM1. In addition, the UCH inhibitor LDN-57444 exhibited specific inhibition of recombinant TsUCH37 and reduced the viability of cultured L1 larvae.

**Conclusions:**

This study reports the identification of the first *T. spiralis* DUB, a cysteine protease that is putatively orthologous to the human protein, hUCH-L5. Results suggest that the interaction of this protein with the proteasome has been conserved throughout evolution. We show potential for the use of inhibitor compounds to elucidate the role of UCH enzymes in *T. spiralis* infection and their investigation as therapeutic targets for trichinellosis.

## Author Summary

*Trichinella spiralis* is a parasitic nematode that infects mammals indiscriminately. Although the biggest impact of trichinellosis is observed in developing countries, the parasite is found on all continents except Antarctica. In humans, *Trichinella* infection contributes globally to helminth related morbidity and disability adjusted life years. In animals, infection is implicated as a serious agricultural problem and drug treatment is largely ineffective. During chronic infection, larvae invade skeletal muscle cells, forming a nurse cell complex in which they become encysted. The nurse cell is a product of the severe disruption of the host cell homeostasis. Proteins of the Ub/proteasome pathway are highly conserved throughout evolution, and considering their importance in the regulation of cell homeostasis, provide interesting and novel therapeutic targets for various diseases. In order to target this system in parasites, pathogen proteins that play a role in this pathway must be identified. We report the identification of the first *T. spiralis* deubiquitinating enzyme, and show evidence that the function of this protein as a proteasome interaction partner has been evolutionarily conserved. We show that members of this enzyme family are important for *T. spiralis* survival and that the use of inhibitor compounds may help elucidate their role in infection.

## Introduction

*Trichinella spiralis* is a parasitic nematode that infects mammals indiscriminately. Human infection is found on all continents except Antarctica, contributing globally to helminth related morbidity and disability adjusted life years [1]. Perhaps more importantly, this zoonotic, food-born parasite has been implicated as a serious agricultural problem, as even with current screening methods of livestock for market, transmission commonly occurs from sylvatic animals [2]. Unlike the majority of parasitic helminths, *T. spiralis* parasites will only survive a direct host-to-host transmission, modulating host immunity and normal cellular function at all stages of infection [3], [4].

L1 larvae are ingested in contaminated meat and released from infected muscle tissue following digestion. The parasites mature and reproduce in the small intestine, causing symptoms similar to more common intestinal infections, leading to the frequent misdiagnosis of the condition [5]. Female adult worms release newborn larvae that cross the intestinal epithelia and enter the circulation, in which they are transported to skeletal muscle. The number of larvae ingested and their path taken en route to the skeletal muscle tissues determines the potential for acute illness, whereby infection and inflammation of the eyes, heart or CNS may result in myalgia, vasculitis, myocarditis and encephalitis, occasionally leading to fatality [6], [7]. In order to establish chronic infection, parasites invade fully differentiated skeletal myofibres. Here, the phenotype of the host cell is dramatically modified, undergoing de-differentiation, cell cycle arrest and the formation of a surrounding collagen capsule [8]. This parasite infected, multinucleated cell has been termed the nurse cell complex, a structure in which the worm may reside for decades. The disruption of host cell signalling during this process is most likely mediated by *T. spiralis* surface proteins and proteins secreted from the parasite secretory organelle, the stichosome. Only a small proportion of these proteins have been fully characterised [9]. In order to enhance our understanding of this unique process, identifying proteins expressed by *T. spiralis*, and the systems in which they function, is crucial if we are to highlight potential new targets for treatment of the associated pathogenesis.

Current treatment of trichinellosis largely involves the use of wide-spectrum anthelmintics that are effective on parasitic worms whose infection is confined to the intestine of the host [10]. The success of these drugs in trichinellosis is therefore restricted to the early stages of infection during the maturation and reproduction of adult parasites in the gut. As early as 5-7 days after ingestion, the newborn *T. spiralis* larvae enter the circulation where successful treatment now relies on intestinal absorption of the drug. General anthelmintics such as benzimidazole and its derivatives show poor solubility and therefore low efficacy in treatment of migratory stage and muscle stage *T. spiralis* [11], [12]. The prevention or treatment of acute host inflammatory responses to migrating larvae, and the inhibition of nurse cell formation, requires the investigation of new drugs that show improved efficacy, reducing the risk of transmission to a subsequent host.

The ubiquitin pathway is necessary throughout all stages of eukaryotic cell development. The dynamic modification of a substrate protein with ubiquitin (Ub) can modify its function, localization and fate in the cell [13]. Ub conjugation relies on a cascade of enzymes, and its removal is mediated by deubiquitinating enzymes (DUBs), the majority of which are cysteine proteases. Understanding the function of Ub hydrolases in immunology and infection has become of increasing interest due, in part, to their discovery in systems that lack endogenous Ub/proteasome machinery [14]–[16] and in parasitic eukaryotes [17], [18]. This has led to a number of interesting hypotheses regarding the function of these proteins in pathogen-host interactions. It has been shown that viral proteins are able to disrupt the host Ub pathway in a number of ways [19]. For example, the Epstein Barr virus protein EBNA1 directly interacts with the human DUB HAUSP, which plays a role in p53 stability and therefore cell cycle regulation [20]. In most cases there is not yet evidence to show whether or not these interactions extend to pathogen-derived DUBs recognising host proteins as substrates.

In this study, we describe the use of activity-based probes to identify DUBs in *T. spiralis* worm extract of L1 larvae collected from nurse cell complexes. We identify and clone the predicted gene encoding TsUCH37, the putative UCH-L5 homologue, and find evidence that the association of this protein with the proteasome has been conserved throughout evolution. We show that recombinant TsUCH37 activity can be specifically inhibited by a UCH inhibitor, LDN-57444, and that the same drug causes a reduction in viability of parasites in culture. This family of enzymes in *T. spiralis* may be important for the survival of the parasite and similar inhibitory compounds may provide useful tools for further investigating the role of the Ub/proteasome system in *T. spiralis* infection.

## Materials and Methods

### Ethics statement

All procedures involving care and maintenance of animals were approved by the Imperial College Ethical Review Committee and performed under license from the UK Home Office.

### Parasite isolation and culture

*T. spiralis* parasites were maintained in female Sprague-Dawley rats and muscle larvae were isolated from infected rats by digestion of skeletal muscle with acidified pepsin. Parasites were cultured in sterile RPMI (Gibco) supplemented with 1% w/v glucose, 100 U/ml penicillin, 100 µg/ml gentamycin, 20 U/ml nystatin and 2 mM glutamine.

### Parasite lysis

*T. spiralis* parasites were lysed using NP-40 lysis buffer (50 mM Tris-HCl pH 7.4, 150 mM NaCl, and 1% NP-40) and dounce homogenisation. Lysate was cleared by centrifugation at 16000 xg and supernatant protein concentration was determined by BCA assay (Pierce). The reducing agent dithiothreitol (DTT) was added (1 mM) to lysates used in probe reactions.

### Probes

N-ethylmalemide (NEM, an irreversible cysteine protease inhibitor) was added (2 mM) to the control lysate sample and incubated at room temperature for 20 minutes. HA-Ub-VME probe, generated as previously described [21], was then added to all lysate samples at 0.3 µg per 20 µg parasite protein and allowed to react for 1.5–2 hours at room temperature. Samples were boiled in SDS-PAGE loading buffer and separated by reducing gel electrophoresis.

### Immuno-blot analysis

Proteins were transferred onto PVDF membrane and blocked for 1 hour at room temperature in 5% w/v non-fat milk with PBS-Tween (0.1% v/v). Membranes were then incubated in anti-HA-HRP (Roche) in 2% non-fat milk with PBS-Tween (0.1%). Membranes were washed in PBS-Tween (0.1%) before being visualised using enhanced chemiluminescence substrate (PerkinElmer).

### HA immuno-precipitation

Probe reactions were carried out as described with *T. spiralis* L1 larvae lysate (DTT free). SDS was added (0.4% w/v) and each sample was vortexed before diluting out the SDS to 0.1% w/v with wash buffer containing 0.1% v/v NP-40, 50 mM Tris-HCl pH 7.4, 150 mM NaCl. Wash buffer-equilibrated Protein G sepharose beads (SIGMA) were used to pre-clear samples at 4°C for 2 hours. Anti-HA affinity matrix (Roche) was then added to each sample (30 µl beads per ml of lysate) and incubated at 4°C overnight. Beads containing bound enzyme-probe complexes were isolated and washed in wash buffer before being boiled in SDS page loading buffer (buffer: beads, 2∶1 volume ratio) to elute protein. Samples were resolved using SDS-PAGE gel electrophoresis and visualised using colloidal Coomassie staining.

### Protein gel extraction and LC/MS/MS analysis

Protein bands were manually excised from colloidal Coomassie-stained SDS-PAGE gels. Each band was digested with trypsin into polypeptide fragments of approximately 8 to 20 amino acids in length. These were submitted to LC/MS/MS analysis. Spectra obtained were analysed using two independent algorithms, Mascot [22] and Sequest [23], and matched against protein or nucleotide sequences from three different databases: a non-redundant peptide sequence database, an EST invertebrate database and an EST nematodes database. EST matches were analysed further by BLASTx homology searches (nr_FASTA protein database, NCBI).

### Cloning

Pelleted *T. spiralis* L1 larvae were frozen in liquid nitrogen before being shattered in a percussive disruptor. Samples were thawed and total RNA was extracted using the RNeasy kit (Qiagen). The AUGUSTUS [24] algorithm predicted ORF of TsUCH37 (modelled on the sequence of the *T. spiralis* contig 1.2 from bases 35557 to 36904) was amplified using the One Step RT-PCR kit (Qiagen). Primers (5′-tgcaGGATCCatggctgaaggaaattggtgtttaa and 3′-acagtGCGGCCGCttattcaaagacgaaatcatgtgcaa) ending with BamHI and NotI linkers (shown in capitals) respectively, were used to amplify a 931 bp fragment corresponding to the predicted TsUCH37 ORF. The resulting PCR product was cloned between BamHI and NotI sites in pGEM-Teasy (Promega) and sequence verified (Beckman-Coulter). The insert was moved from pGEM-Teasy to pPET28a(+) (Novagen) expression vector between BamHI and NotI sites to ensure TsUCH37 was in-frame with an N-terminal HIS (x6) tag. Amplification of a 313 bp fragment (121-434, primers: 5′-cgactgggtcctatgcctct and 3′-tttcggtgtggttgtgtcgg) of *T. spiralis* GM2 activator protein from total *T. spiralis* RNA was carried out as a control [25].

### Recombinant protein expression and purification

pPET28a(+) vector containing the TsUCH37 insert was transformed into E. coli Rosetta-2 (BL21 derivatives, Novagen) and protein expression was induced by 0.5 mM IPTG for 3 hours at 37°C before being harvested. Cell pellets were lysed using BugBuster™ protein extraction reagent (Novagen) supplemented with lysozyme and DNase I and cleared by centrifugation for 20 minutes at 16000 xg at 4°C. Soluble and insoluble fractions were separated and analysed by SDS-PAGE. HIS-tagged recombinant protein was purified from inclusion bodies on Ni-NTA resin (QIAGEN) under denaturing conditions according to the manufacturers protocol. Recombinant protein was refolded in wash buffer.

### Ub-AMC activity assays

Recombinant *T. spiralis* TsUCH37 was used at between 12.5 nM and 2 µM and Ub C-terminal 7-amido-4-methylcoumarin (Ub-AMC, Boston Biochem) was used at 250 nM, both in reaction buffer (150 mM NaCl, 50 mM Tris/HCl pH 7.5, 2 mM EDTA, 2 mM DTT supplemented with 1 mg/ml bovine serum albumin) at room temperature. Negative control samples were incubated with 2 mM NEM for 20 minutes before addition of Ub-AMC substrate. Purified PfUCHL3 (74 nM) was used as a positive control for Ub-AMC hydrolysis [17]. AMC cleavage was measured by fluorescence at 368 nm excitation and 467 emission wavelengths on a FLUOstar microplate reader (BMG LABTECH). All measurements were made in a 384 well plate (Nunc, black, Thermo Scientific) in a time dependent manner. Reactions were carried out in a total volume of 20 µl in triplicate and all protein concentrations were measured using the BCA assay.

### Co-precipitation

*T. spiralis* L1 larvae were lysed in NP-40 lysis buffer and concentrations were determined by BCA assay. Lysate (3 mg per sample) was pre-cleared using wash buffer-equilibrated-Protein G sepharose beads (SIGMA) at 4°C for 2 hours. Recombinant native TsUCH37 (500 µg) was bound to Ni-NTA resin in binding buffer (1xPBS pH 8.0, 300 mM NaCl, 0.1% NP40, 50 mM imidazole and 2 mM 2-mercaptoethanol) for 2 hours at 4°C. Pre-cleared *T. spiralis* lysate was incubated with either TsUCH37-bound Ni-NTA resin or native Ni-NTA (control) overnight at 4°C. Beads were washed 3 times in binding buffer and proteins that were bound to the beads were separated by SDS-PAGE and analysed by LC/MS/MS as described.

### UCH inhibitor

LDN-57444 (SIGMA) was solubilised in DMSO and used at concentrations between 10 µM and 1 mM as indicated in the results.

### MTT viability assay

Parasite viability was measured by a quantitative colorimetric assay with the tetrazolium salt 3-[4,5-diethylthiazol-2-yl]-2,5-diphenyltetrazolium bromide, MTT (SIGMA) [26]–[28]. Following drug treatment in culture, 1000 *T. spiralis* L1 larvae were incubated in 5 mg/ml MTT in phenol red free RPMI 1640 (SIGMA) for 4 hours at 37°C. Formazan crystals formed in viable larvae were solubilised by shaking in 200 µl of 100% DMSO for 1 hour at room temperature. Parasites were removed and the absorbance of the supernatant at 575 nm was determined. Parasites that had been killed by heat treatment (65°C for 10 minutes) were used as a negative control for absorbance at 575 nm. All measurements were made in triplicate. Data was statistically analysed by a two-way ANOVA test.

## Results

### Deubiquitinating activity is present in *T. spiralis* L1 larvae

**Figure 1:**
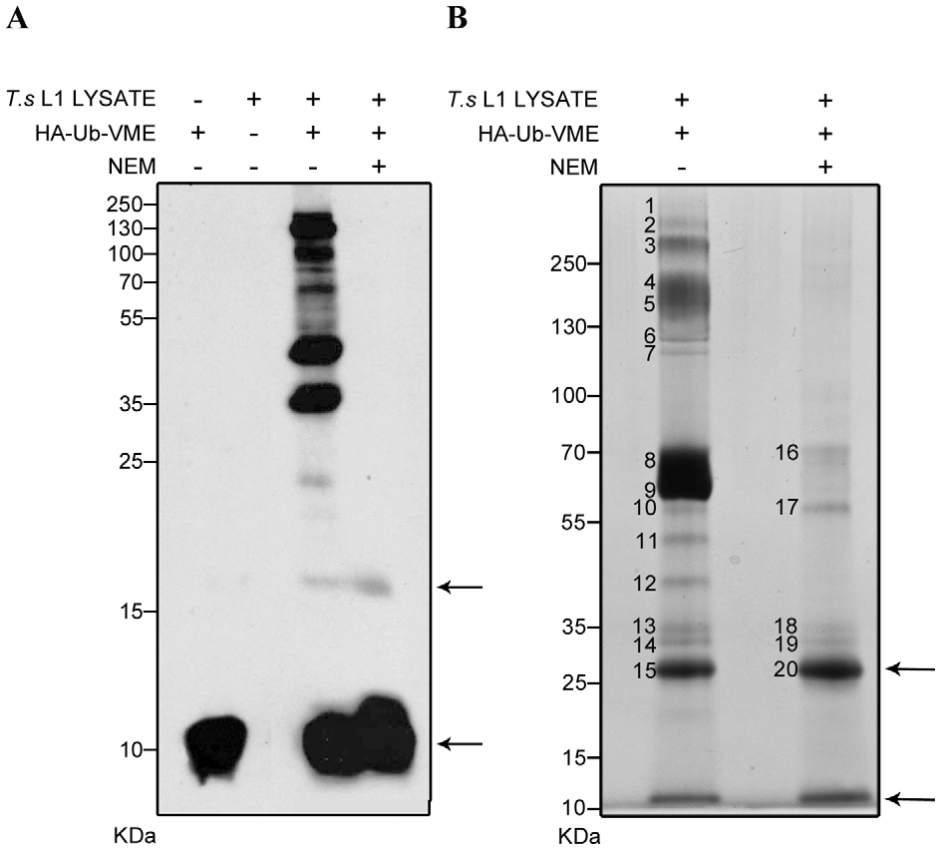
Identification of deubiquitinating activity in *T. spiralis* L1 lysate. (A) Lysate of *T. spiralis* L1 larvae was reacted with HA-Ub-VME, separated by 10% SDS-PAGE and analysed by immuno-blot using anti-HA-HRP. Reactions were carried out either in the absence or presence of NEM in order to determine cysteine specificity. Probe alone and lysate alone were included as controls (left hand lanes). Arrows indicate un-reacted probe or probe aggregates and molecular weights are shown in kDa. (B) Large-scale immuno-precipitation was carried out to isolate *T. spiralis* protein-HA-Ub-VME complexes on an anti-HA affinity matrix. Samples were separated by SDS-PAGE and 20 bands (numbered) were excised and analysed by tandem mass spectrometry. Arrows indicate the anti-HA antibody light chain (25 kDa) and un-reacted probe (10 kDa).

HA-Ub-VME is a haemmaglutinin-tagged probe whose C-terminal electrophilic group (VME) forms a covalent thioether bond with the active site cysteine of a DUB [21]. DUB enzymes specifically recognise the Ub moiety as their substrate, and in an attempt to cleave it, become irreversibly bound to the probe. In order to identify DUB activity in proteins expressed by *T. spiralis*, lysate of L1 larvae (containing proteins from both the secretory organelle, the stichosome, and non-secreted proteins) was reacted with the HA-Ub-VME probe that was derived from human Ub, which shows 97.4% identity to the putative *T. spiralis* Ub. Reactivity was analysed by immuno-blot (anti-HA, Figure 1A) either in the presence or absence of NEM. HA-Ub-VME probe alone was found to yield a band at its expected size of 10 kDa and a second, less prominent band at 20 kDa that may correlate with probe aggregation. A number of reactive bands were observed after incubation of *T. spiralis* lysate with the Ub probe and immuno-blotting with anti-HA-HRP. This reactivity was eliminated by pre-incubation with NEM, denoting cysteine dependent activity. The most prominent cysteine specific bands showed electrophoretic mobility corresponding to proteins with molecular weights within the 30–150 kDa range. No non-specific reactivity was observed in samples containing lysate alone.

### Putative DUB homologues are expressed by *T. spiralis* L1 larvae

In order to further characterise proteins with DUB activity expressed by *T. spiralis*, lysate of L1 larvae was reacted on a preparative scale with HA-Ub-VME in the presence or absence of NEM. Protein-probe complexes were then immuno-precipitated on immobilised anti-HA affinity matrix and analysed by SDS-PAGE (Figure 1B). Protein-probe complexes were visualised by Coomassie staining, revealing 15 bands between 27 and >250 kDa from the NEM negative sample and five bands between 27 and 70 kDa from the NEM positive sample. Reducing conditions caused the dissociation of the anti-HA antibody light chain from the affinity beads, which showed as expected, a band corresponding to a protein of approximately 25 kDa. A most prominent band was observed between 60 and 70 kDa of which no clear product was seen to correspond in the immuno-blot analysis of worm lysate protein-probe complexes. 20 bands from the Coomassie-stained gel were manually excised and peptide fragments produced by trypsin digestion were analysed using LC/MS/MS. Spectra were analysed by both Mascot and Sequest (independently) and matched against protein or nucleotide sequences from 3 different databases: 1) a non-redundant peptide sequence database (nr_FASTA: NCBI-All non-redundant GenBank CDS translations + RefSeq Proteins + PDB + SwissProt + PIR + PRF), 2) an EST invertebrate database (est Database of GenBank + EMBL + DDBJ sequences from EST Division: est_others) and 3) an EST nematodes database (EST Division: est_nematdoes).

The genome of *T. spiralis* was only very recently released in a draft form [29]. Therefore at the time these experiments were initiated, the most reliable source of data for our spectral matches was derived from the *T. spiralis* EST database. Each search identified a number of putative DUB proteins or EST nucleotide sequences that matched peptides found in the 15 bands excised from the NEM negative sample of the gel. No DUB-related matches were found in the NEM positive samples. Un-annotated nematode EST sequence matches were further analysed by BLAST homology searches. In total, sequences homologous to five human DUBs: UCH-L5, UCH-L3, HAUSP, OTU 6B and Ataxin-3 were identified. A comprehensive list of DUB-specific results can be found in Table 1. The significance of a match was determined based on three criteria: 1) matches that were found in more than one database search with a large number of unique peptides, 2) matches that were identified in both Sequest and Mascot analysis and 3) multiple matches that gave the same BLAST homology search results. Hits also included a match to the putative *T. spiralis* ADRM1 proteasome subunit, putative *T. spiralis* Ub and polyubiquitin and matches to common contaminant proteins such as keratin and serum albumin, the latter being found most abundantly in band 9.

**Table 1:** LC/MS/MS and BLAST analysis of HA-Ub-VME reactive *T. spiralis* proteins.

						HIGHEST MASCOT SCORE		HIGHEST SEQUEST SCORE	
BAND EXCISED	EST Genbank ID	SPECIES (EST)	BLASTx MATCH	HUMAN HOMOLOGUE	CONSERVED DOMAIN	No. of unique peptides	Total no. of spectra	Score	Total No. Peptides
1,2,4,5,6,7,15	157958881	*T. spiralis*	UCH-L5 *M. musculus*	UCH-L5	Peptidase C12	2	7	60.27	73
5,6,7,11,12,13,15	148305659	*T. spiralis*	UCH-L5 *M. musculus*	UCH-L5	Peptidase C12	7	37		
13,4	13182314	*T. spiralis*	OTU domain protein 6B *T. spiralis*	OTU 6B	OTU	1	1	10.24	2
1,3,5,6,8,11,14	18684491	*Parastrongyloides trichosuri*	OTU cysteine protease B. malayi	OTU 6B	OTU			10.24	6
5,12,13,14	148302713	*Gallus gallus*	Ubiquitin c-terminal hydrolase family 1 *T. spiralis*	UCH-L3	Peptidase C12	4	15	20.36	5
6	13182064	*T. spiralis*	Ubiquitin c-terminal hydrolase 7 *T. spiralis*	HAUSP	MATH, Peptidase C19	4	5	60.17	15
5	158008116	*A. caninum*	Ubiquitin c-terminal hydrolase 7 *A. caninum*/USP7 *HSV*	HAUSP	Peptidase C19	2	2		
1, 3, 4, 5, 6	117581976	*T. spiralis*	Ubiquitin-specific protease like protein	HAUSP	MATH, Peptidase C19	3	2	130.2	24
1	5555824	*C. elegans*	ATAXIN-3 *C. elegans*	ATX-3	JOPSEPHIN			10.18	4
10	14017173	*T. spiralis*	ADRM1 *T. spiralis*	ADRM1	Proteasome Rpn13	1	1	20.25	2

LC/MS/MS data was analysed by both Mascot and Sequest searches of nr_FASTA, EST_invertebrates and EST_nematodes databases. BLAST homology searches were carried out to identify conserved domains and protein orthology. Table shows a list of all DUB related matches found in protein samples numbered in Figure 1B.

The most significant result, with a large number of peptide matches (and therefore the greatest percentage coverage equivalent) was found predominantly in bands 11, 12, and 13. Here, matches were identified as two *T. spiralis* cDNA EST fragment sequences with the accession numbers, GenBank gi148305659 and gi157958881. Each match was analysed by a BLASTx homology search (translated nucleotide to protein) yielding the mouse Ub C-terminal hydrolase UCH-L5 protein as the first hit. The Homo sapiens UCH-L5 protein was observed as being homologous to gi148305659 and gi157958881 to e-values of 8×10^−63^ and 6×10^−63^ respectively. The translated EST sequences contain a conserved peptidase C12 domain, which is found in UCH-L5 homologues and comprises four conserved specific catalytic amino acids at its active site, glutamine, cysteine, histidine and aspartic acid. The central cysteine residue is responsible for the initiation of the cleavage of Ub from a substrate protein. The predominant location of peptides matching this protein (in the 35–50 kDa region) corresponded approximately to the size of the human UCH-L5 protein (37 kDa) in complex with a 10 kDa probe, however matches to UCH-L5 were also found in all other bands of the NEM negative lane except bands 3, 8, and 9 possibly due to aggregation or oligomerisation.

### Recombinant *T. spiralis* TsUCH37, a putative UCH-L5 homologue, has deubiquitinating activity

**Figure 2:**
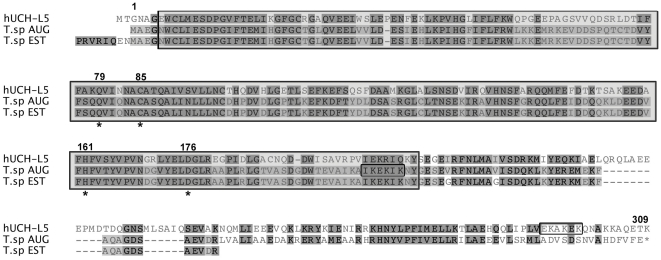
Protein alignment (MUSCLE) of the translated putative *T. spiralis* TsUCH37 sequence, EST gi157958881 and human UCH-L5 protein sequence. The translated ORF sequence generated by AUGUSTUS (T.sp AUG) was aligned with the translated EST gi157958881 (T.sp EST) and human UCH-L5 (hUCH-L5) protein sequence (Drummond AJ et al, www.geneious.com). The full peptidase C12 domain (shaded box) and catalytic residues (asterisk) of hUCH-L5 are indicated. The TsUCH37 AUGUSTUS sequence displays 45% identity and 16% homology to hUCH-L5 and has a predicted molecular weight of 35.22 kDa. Conserved residues are highlighted where identical and homologous. Positions of the putative TsUCH37 start methionine, catalytic residues and the predicted end of the protein are numbered. The KEKE domain of the hUCH-L5 sequence and a potential KEKE domain of the putative TsUCH37 sequence have been outlined.

Due to its significantly high score, we decided to further characterise the putative UCH-L5 homologue in *T. spiralis*. The complete draft assembly contig database was available from The Genome Institute at Washington University (accessed August 2010) [30]. The longer of the two *T. spiralis* ESTs that matched UCH-L5 sequences, gi157958881, was found to align well with contig 1.2 between bases 35557 and 36904. Contig 1.2 was analysed by 4 different gene prediction programmes: AUGUSTUS [24], GenemarkHMM [31], SNAP [32] and Fgenesh [33]. In addition, two of these algorithms were used to analyse the sequence with extra constraints: AUGUSTUS with the EST gi157958881 and/or the C. elegans nematode UCH-L5 orthologue (UBH-4) as anchors, Fgenensh_C using the EST as an anchor and Fgenesh+ with UBH-4 as an anchor. This gave 7 potential open reading frames (ORF) for the gene (Figure S1). When analysed by BLAST homology searches, all sequences spanned the conserved peptidase C12 domain. Of the 7 predictions, 5 gave the same start codon, that which was already present in the EST gi157958881, whereas the Fgenesh de novo and Fgenesh_C predicted a gene twice as long. This portion of the *T. spiralis* draft genome was also annotated using the Fgenesh algorithm, predicting an open reading frame containing 2 protein domains, with the peptidase C12 domain and an additional upstream PRP38 domain [29]. Each predicted sequence was translated into protein and aligned with a number of putatively orthologous UCH-L5 coding sequences (data not shown). This revealed that the AUGUSTUS prediction aligned more closely with the consensus sequence of a multitude of putative orthologues (Figure S2). Alignments show that the peptidase C12 domain of these proteins is located very close to the N-terminus. The AUGUSTUS prediction, a 930 bp open reading frame of 7 exons, contained a start codon and peptidase C12 domain that when translated, aligned closely with the human UCH-L5 protein sequence (Figure 2). All 4 catalytic residues typical to the UCH family of DUBs also aligned. Orthologous UCH-L5 proteins, including that expressed in the C. elegans nematode, do not contain an additional upstream PRP38 domain and no peptides isolated by LC/MS/MS matched a PRP38 domain in the nr_FASTA or the EST databases. For these reasons, primers were designed based on the AUGUSTUS gene prediction in order to clone the predicted TsUCH37 sequence from *T. spiralis* cDNA.

**Figure 3:**
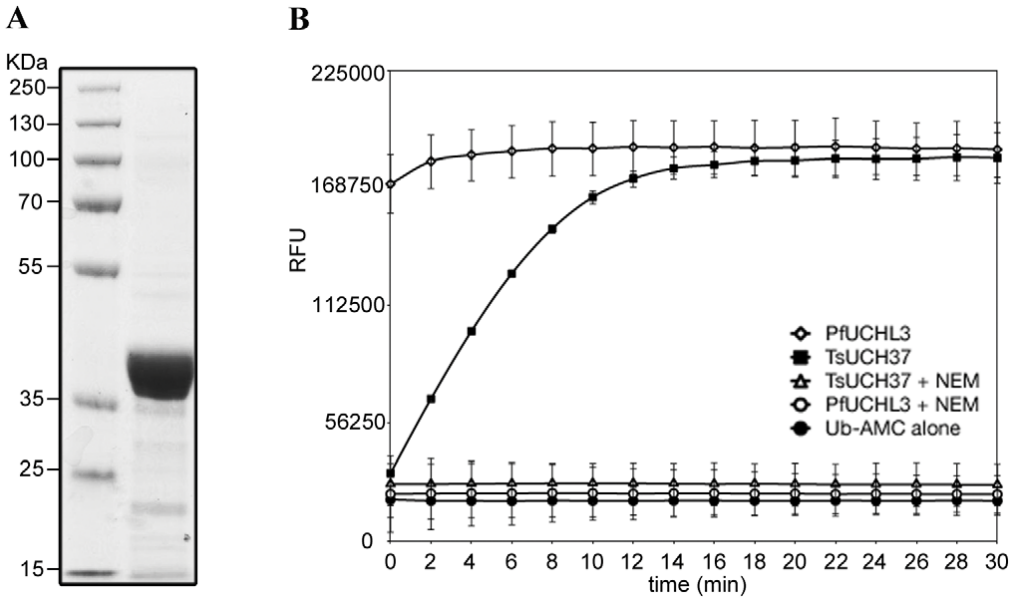
Expression, purification and verification of the deubiquitinating activity of recombinant TsUCH37. (A) HIS-tagged recombinant TsUCH37 was expressed and purified yielding a protein of approximately 36 kDa when separated by SDS-PAGE. (B) Recombinant TsUCH37 (2 µM) was reacted with Ub-AMC. Ub-AMC hydrolysis was measured in RFU as fluorogenic AMC was released over time (minutes). All assays were carried out in triplicate. Points show the mean fluorescence with standard deviation (error bars are indicated). Deubiquitinating activity was fully inhibited by the pre-incubation of the protein with NEM. Plasmodium falciparum PfUCH-L3 (74 nM) was used as a positive control for deubiquitinating activity.

The recombinant TsUCH37 protein was expressed with an N-terminal HIS tag in E. coli. The purified protein from insoluble fraction was analysed by SDS-PAGE, showing a protein of approximately 36 kDa in size (Figure 3A). To verify that the recombinant TsUCH37 protein possesses true deubiquitinating activity, Ub conjugated to 7-amino-4-methylcoumarin (Ub-AMC) was used to measure its enzyme activity. DUB proteins cleave the Ub-AMC substrate, releasing fluorogenic AMC, the measurement of which corresponds to enzyme activity. Purified recombinant TsUCH37 (2 µM) was reacted with Ub-AMC (250 nM) either with or without pre-incubation with NEM (Figure 3B). Fluorescence corresponding to Ub cleavage was measured at two-minute intervals for a duration of 30 minutes following the addition of the substrate. Purified Plasmodium falciparum UCHL3 was assayed in saturating concentrations (74 nM) as a positive control as it has been previously reported to possess good deubiquitinating activity [17]. The reaction showed Ub cleavage by recombinant TsUCH37 within 2 minutes following addition of the substrate, indicating physiologically relevant activity (rather than end-point as indicated by reactivity to probe). This activity was silenced by pre-incubation with NEM, verifying the necessity for a functional cysteine residue for substrate cleavage.

### Recombinant TsUCH37 associates with putative *T. spiralis* proteasome components

**Figure 4:**
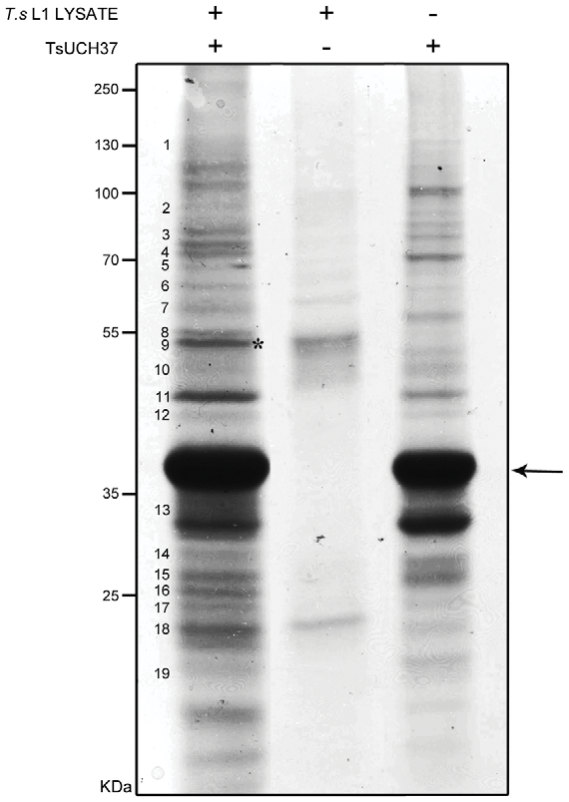
Identification of recombinant TsUCH37 associated *T. spiralis* proteins. Ni-NTA bound TsUCH37 (with an N-terminal HIS-tag) was incubated with pre-cleared *T. spiralis* L1 larvae lysate. Associated proteins were isolated by co-precipitation and separated by SDS-PAGE. Samples containing native Ni-NTA resin incubated with pre-cleared *T. spiralis* lysate (middle lane) and Ni-NTA bound recombinant TsUCH37 incubated with lysis buffer (right lane) were included as controls. The gel was visualised using colloidal Coomassie staining and 19 (numbered) bands were excised and analysed by LC/MS/MS. Recombinant HIS-tagged TsUCH37 is indicated by an arrow and the location of peptides matching the putative *T. spiralis* ADRM1 protein sequence is marked with an asterisk. Molecular weights are shown in kDa.

The human UCH-L5 and yeast UCH37 (YUH1) associate with the 26S proteasome complex [34], [35]. They do so via the interaction of a KEKE motif, located on the C-terminal tail of the protein, with the ADRM1 (human) or Rpn13 (yeast) subunit of the proteasome. To assess whether this may also be the case for TsUCH37 in *T. spiralis*, we looked for proteins from lysate of L1 larvae that associated with HIS-tagged recombinant TsUCH37. TsUCH37-associated proteins were separated by SDS-PAGE and subjected to LC/MS/MS (Figure 4). A number of proteins were observed after reaction of lysate with the recombinant TsUCH37. These proteins were not present in either the sample containing recombinant TsUCH37 alone or the lysate incubated with Ni-NTA beads alone. These were manually excised from the gel, analysed by LC/MS/MS, and data was matched against both the nr_FASTA protein database and the EST_nematodes database using Mascot. Peptides matched EST sequences found to be homologous to 12 different putative *T. spiralis* proteasome components of both the 19S and the 20S subunits, including putative homologues of the regulatory Rpn 1 and 2 subunits, and components of the proteasome core ATPase. Table 2 shows a list of all the matches corresponding to *T. spiralis* proteasome components. The putative *T. spiralis* ADRM1 protein sequence (yeast Rpn13 homologue) was also identified, the same sequence identified previously by HA-Ub-VME immuno-precipitation.

**Table 2:** Spectra were searched against EST_nematodes and nr_FASTA databases using Mascot.

						MASCOT SCORE		
BAND	EST/put. protein ID	SPECIES (EST)	BLASTx DESCRIPTION-*T. spiralis* put. proteasome component	HUMAN HOMOLOGUE	CONSERVED DOMAIN	No. of unique peptides	Total no. of spectra	Molecular weight (kDa)
1	157957916	*T. spiralis*	Rpn-2	26S non-ATPase regulatory subunit 1	RPN2	2	2	55
2	148306182	*T. spiralis*	26S proteasome non-ATPase regulatory subunit 2	26S proteasome non-ATPase regulatory subunit 2	RPN1	3	4	56
7	316974493	*T. spiralis*	26S proteasome non-ATPase regulatory subunit 3	26S proteasome non-ATPase regulatory subunit 3	RPN3	6	7	59
8	148300883	*T. spiralis*	26S protease regulatory subunit 4	Proteasome ATPase subunit 4	P-loop NTPase, RPT2	2	2	58
9	316973936	*T. spiralis*	26S protease non-ATPase regulatory subunit 5	26S proteasome non-ATPase regulatory subunit 4	Rpn10	6	6	45
9	316977948	*T. spiralis*	ADRM1	ADRM1	Rpn13	2	2	46
10	157958515	*T. spiralis*	Rpt-5	Proteasome ATPase subunit 3	P-loop NTPase, RPT5	2	2	58
10	148301308	*T. spiralis*	26S proteasome non-ATPase regulatory subunit 11	26S proteasome non-ATPase regulatory subunit 11	RPN6	2	2	65
11	316968151	*T. spiralis*	26S proteasome regulatory subunit 6B	26S protease regulatory subunit 6B	RPT3	4	4	39
12	316972496	*T. spiralis*	26S proteasome regulatory subunit 8	26S protease regulatory subunit 7	RPT1	4	4	39
14	157958781	*T. spiralis*	Putative proteasome activator complex subunit 3	PSME3	PA28 alpha, PA28 beta	5	17	58
17	148302503	*T. spiralis*	Proteasome activator complex subunit 3	PSME4	PA28 alpha, PA28 beta	4	4	64
19	148307154	*T. spiralis*	Proteasome subunit alpha type-2-A	Proteasome subunit alpha type-2	proteasome alpha type-2	2	2	50

The table lists *T. spiralis* proteasome specific matches to peptides isolated by co-precipitation of lysate with recombinant TsUCH37.

### LDN-57444 specifically inhibits recombinant TsUCH37 deubiquitinating activity and reduces viability of L1 larvae

**Figure 5:**
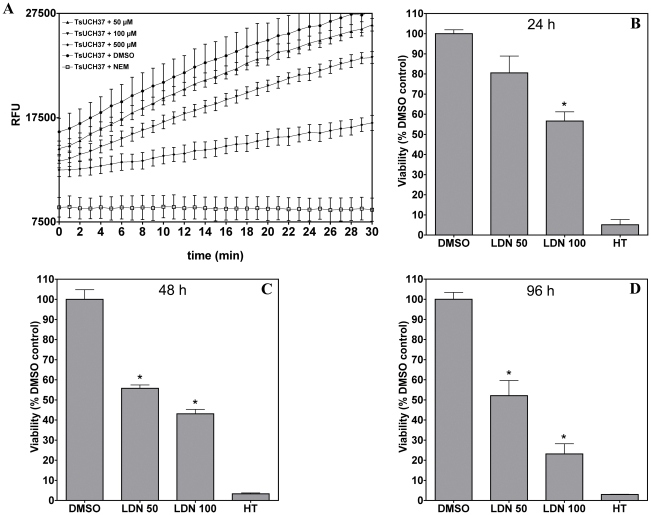
LDN-57444 inhibition of recombinant TsUCH37 DUB activity and parasite viability. Recombinant TsUCH37 was incubated with the UCH inhibitor LDN-57444. Panel A shows the cleavage of Ub from fluorogenic AMC by 12.5 nM TsUCH37 (measured as relative fluorescence units, RFU) after incubation with 50 µM, 100 µM and 500 µM of LDN-57444 solubilised in DMSO. Pre-incubation of TsUCH37 with NEM was included as a negative control and incubation with DMSO alone as a positive control. All assays were carried out in triplicate. Points show the mean fluorescence with standard deviation (error bars are indicated). (B), (C) and (D) The susceptibility of *T. spiralis* L1 larvae cultured in the presence of DMSO or 50 µM and 100 µM LDN-57444 after 24, 48 and 96 hours was measured as absorbance of formazan at 575 nm using the MTT assay. All assays were carried out in triplicate. The mean percentage of the DMSO control (100%) with 1 standard deviation is shown. Baseline absorbance was taken by MTT assay of heat-killed (HT) parasites. Statistically significant data are indicated with asterisks (*, P<0.05 versus parasites incubated with DMSO alone).

LDN-57444, otherwise known as compound 30 (C30), is an isatin o-acyl oxime that exhibits active site-directed inhibition of hUCH-L3 and, with greater potency, hUCH-L1 [36]. Treatment of mammalian neuronal cells with LDN-57444 causes an increase in the levels of highly ubiquitinated proteins [37]. Both hUCH-L1 and hUCH-L3 contain a peptidase C12 domain, utilising the same catalytic triad of amino acids as hUCH-L5 and TsUCH37 to hydrolyse Ub. In order to establish if this compound is capable of TsUCH37 inhibition, recombinant protein (12.5 nM) was incubated with either the drug (solubilised in DMSO) or with DMSO alone, before a measurement of DUB activity was taken in relative fluorescence units using Ub-AMC as the substrate. A titration of the drug concentration (5, 10, 50, 100 and 500 µM) revealed specific inhibition of recombinant TsUCH37. Figure 5, panel A shows the effect of 50, 100 and 500 µM of LDN-57444.

The MTT viability test is a quantitative colorimetric assay based on the tetrazolium salt, 3-[4,5-diethylthiazol-2-yl]-2,5-diphenyltetrazolium bromide [26], [27]. MTT (yellow in colour) is reduced by dehydrogenase enzymes of metabolically active cells to insoluble purple crystals of formazan. Formation of formazan is directly proportional to cell number and is not observed in dead cells. The crystals are solubilised in organic solvents and an absorbance measurement of the culture supernatant is taken, representing cell viability. Over a period of 96 hours, L1 larvae in culture were incubated with either 50 µM or 100 µM of LDN-57444 solubilised in DMSO (Figure 5, panels B, C and D). Larvae were also incubated in DMSO alone giving a control for 100% viability. Heat-killed larvae, producing no formazan, were cultured as a negative control. After 24 hours, a decrease in motility and a change in morphology were observed, with treated larvae becoming uncoiled and stationary. An absorbance measurement of the culture supernatants 24 hours post-treatment, confirmed that larvae incubated with 50 µM LDN-57444 contained 20% less formazan than those incubated in DMSO alone, showing a negative effect on viability. After 96 hours, a 75% reduction in the viability of the parasites treated with 100 µM of the inhibitor was observed.

## Discussion

Only a small number of *T. spiralis* cysteine proteases have been identified and until now none of these had been characterised as Ub hydrolase enzymes [38], [39]. Using small molecule inhibitor probes followed by LC/MS/MS we have identified several potential *T. spiralis* DUBs from lysate containing both the secreted and non-secreted proteins of L1 larvae. Of these, we identified 5 putative protein sequences homologous to the human cysteine protease DUBs: UCH-L5, UCH-L3, HAUSP, OTU 6B and Ataxin-3. We cloned the putative gene for the UCH-L5 homologue and expressed and functionally verified the recombinant protein as the first parasitic nematode DUB, TsUCH37; a C-terminal Ub hydrolase enzyme that shows Ub-AMC cleavage activity with physiological kinetics.

*T. spiralis* UCH37 is a cysteine-dependent deubiquitinating enzyme that contains a Ub C-terminal hydrolase (UCH) domain and is a putative member of the peptidase CA clan. Classification of DUB enzymes is based on this conserved catalytic peptidase domain [40]. The human UCH-L1, L3, L5 and BAP1 (BRCA1 associated protein 1) all contain a peptidase C12 domain, which includes 4 catalytic residues shown to be responsible for cysteine-dependent deubiquitinating activity. Alignment of the translated putative TsUCH37 ORF with orthologous sequences shows that the protein shares 45% amino acid identity with the human UCH-L5 and 43% with the C. elegans UBH-4. Residues 5-209 of the parasite protein comprise the full peptidase C12 domain (Figure 2).

Studies on mammalian UCH-L5 have shown that in HeLa and HEK 293T cells, this enzyme is located in both the cytoplasm and the nucleus [34], [41]. Although a larger proportion of the UCH-L5 is found in association with the 19S (PA700) proteasome subunit, some also exists in a free unassociated state. It has been shown that upon interaction with the 19S subunit of the proteasome, hydrolysis of mono-Ub (as Ub-AMC) is elevated, and hydrolysis of di-Ub becomes possible [34]. This is perhaps due to the change in conformation of a C-terminal tail that sterically blocks the active site in the enzyme's free form [42]. This interaction with the proteasome is mediated by the binding of a KEKE motif found at the C-terminal tail of the DUB with a complementary motif on the C-terminal tail of the proteasome component ADRM1 (yeast Rpn13 homologue) [43]. It is therefore postulated that one major role of UCH-L5 is to hydrolyse Ub from protein substrates as they pass into the 20S core of the proteasome for degradation. At the C-terminal end of the TsUCH37 catalytic domain, albeit upstream of the putative human orthologue, a potential KEKE motif can be found that may correspond to that found at the C-terminus of hUCH-L5 (Figure 2). In addition, a putative homologue for ADRM1 is annotated in the *T. spiralis* genome. The protein sequence for TsADRM1 matched peptides identified by LC/MS/MS from HA-Ub-VME-reacted lysate, suggesting an association between TsADRM1 and probe-bound DUBs. Furthermore, co-precipitation experiments with proteins from L1 larvae lysate using recombinant N-terminally HIS-tagged TsUCH37 as bait, yielded numerous putative *T. spiralis* proteasome components including TsADRM1. This suggests that a specific interaction between UCH37 and ADRM1 occurs and we can hypothesise that this role of TsUCH37, releasing Ub from proteins targeted for degradation, has been conserved throughout evolution.

Due to the complexity of the parenteral phase of trichinellosis, successful treatment of the symptoms that arise in the CNS, cardiac and vascular tissue during parasite migration to the host skeletal muscle, largely relies on the use of corticosteroids [5], [44], [45]. Evidence also suggests that currently used anthelmintics show low efficacy in the management of encysted larvae, and that any effect may be accounted for by the prevention of nurse cell formation rather than the clearance of complexes that are already present [46]. Improved treatment would require the identification of parasite-specific drug targets and the development of drugs capable of a) inhibiting entry of newborn larvae into the circulation, b) targeting migratory larvae in the circulation or c) preventing or clearing encysted larvae. Developing antiparasitic compounds that target these more advanced stages of infection would reduce the risk of medical complications and fatalities and may also prevent zoonotic transmission.

Previous studies have led to the conclusion that *T. spiralis* are refractory to current methods of genetic manipulation and we therefore cannot comment on the essentiality of TsUCH37 in *T. spiralis* survival. Al-Shami et al recently demonstrated that knock out of murine UCH37 (UCH-L5 homologue) was lethal [47]. The essential nature of many Ub/proteasome system enzymes and their expression throughout all stages of eukaryotic development has led to the investigation of these enzymes as potential drug targets in cancer and in infectious disease [48], [49]. In the case of the DUB enzyme family, sequence identity of orthologous proteins may diverge sufficiently for the development of pathogen specific inhibitors [50]. Liu et al showed that although hUCH-L1 and hUCH-L3 share 52% identity, the UCH inhibitors compound 30 (LDN-57444) and compound 11 demonstrate significantly different potency in their relative inhibition of the two enzymes [36]. This illustrates that using exploratory medical chemistry, UCH enzymes containing the same conserved catalytic domain can be specifically and individually drug-targeted. Figure 2 shows differences in the protein sequences of the human and *T. spiralis* UCH-L5 orthologues that may be exploited in the development of specific inhibitors.

We have shown that the active site-directed inhibitor of hUCH-L1 and L3, LDN-57444, specifically inhibits cleavage of Ub-AMC by recombinant TsUCH37. This correlated with a concentration dependent reduction of the viability of *T. spiralis* L1 larvae in culture. It may therefore be possible to target the *T. spiralis* UCH enzyme family with drugs that could be modified for further specificity. Considering that LDN-57444 shows specificity for hUCH-L1 and L3, proteins for which homologues are likely to be expressed by *T. spiralis*, the reduction in viability of parasites in culture cannot be directly accounted for by TsUCH37 inhibition but rather a more general effect stemming from the interface with the UCH DUB family as a whole. The specific target of LDN-57444 in *T. spiralis* therefore remains unknown, however considering its observed effect on the viability of L1 larvae, it can be hypothesised that UCH DUBs play an important role in parasite biology. Inhibition of DUB enzymes blocks the removal of Ub and Ub chains from substrate proteins. In mammalian cells an accumulation of ubiquitinated proteins can lead to the unfolded protein response and endoplasmic reticulum stress, an effect that in neurons is linked to Parkinson's disease [37]. The use of inhibitor compounds such as LDN-57444 may help elucidate the role of UCH enzymes in the process of infection by investigating their effect on circulating larvae, muscle stage larvae and nurse cell complex formation both *ex* and *in vivo*.

Without further analysis of Ub/proteasome components in *T. spiralis* secreted proteins it can only be postulated that TsUCH37 is involved in endogenous protein turnover. It is of great interest to us to investigate whether or not components of the Ub/proteasome system are secreted by *T. spiralis* and indeed if they are secreted into the nurse cell, in order to determine the role of this pathway in infection. The activity-based approach taken in these studies is currently underway to identify DUBs from only the excretory-secretory elements of the worms, proteins that are likely to be specifically host-targeted.

## Supporting Information

Figure S1 Gene predictions of the putative TsUCH37 ORF. The EST fragment obtained by LC/MS/MS (gi157958881) was aligned with contig 1.2 from the draft assembly of the *T. spiralis* genome. At this location, gene predictions from Fgenesh (de novo or using EST as an hint/constraint as Fgenesh+) span a large region of the contig. However, when employing UBH4 (the C. elegans UCH-L5 orthologue) as a hint, the Fgenesh_C predicted start agrees with all other gene prediction programs employed (AUGUSTUS de novo, AUGUSTUS with EST and/or UBH-4 as constraints, SNAP, Genemark.HMM).
(TIF)

Figure S2 Multiple nucleotide alignments (MUSCLE) of putative UCH-L5 orthologues with the *T. spiralis* contig 1.2 (34000-37000), the AUGUSTUS predicted ORF for TsUCH37 and the EST gi157958881. The AUGUSTUS TsUCH37 sequence, EST gi157958881 and the orthologous UCH-L5 sequences are coding sequence only (no introns). The Contig was obtained from the Genome Institute at Washington University, accessed August 2010. The alignment was generated using Geneious (Drummond AJ et al, www.geneious.com). Blocks indicate nucleotide bases and lines indicate gaps in alignment. Accession numbers for coding sequences in order of appearance: D.m NM_001201752, S.c NM_001181757, A.t NM_105238, C.e NM_063283, X.l NM_001095597, H.s NM_001199263, M.m NM_019562, B.m XM_001895545.
(TIF)
