# Supplementary figures and images for: Characterisation of the Trichinella spiralis Deubiquitinating Enzyme, TsUCH37, an Evolutionarily Conserved Proteasome Interaction Partner
Source: PLoS Negl Trop Dis. 2011 Oct 4;5(10):e1340. doi: 10.1371/journal.pntd.0001340 (PMC3186758; doi:10.1371/journal.pntd.0001340)

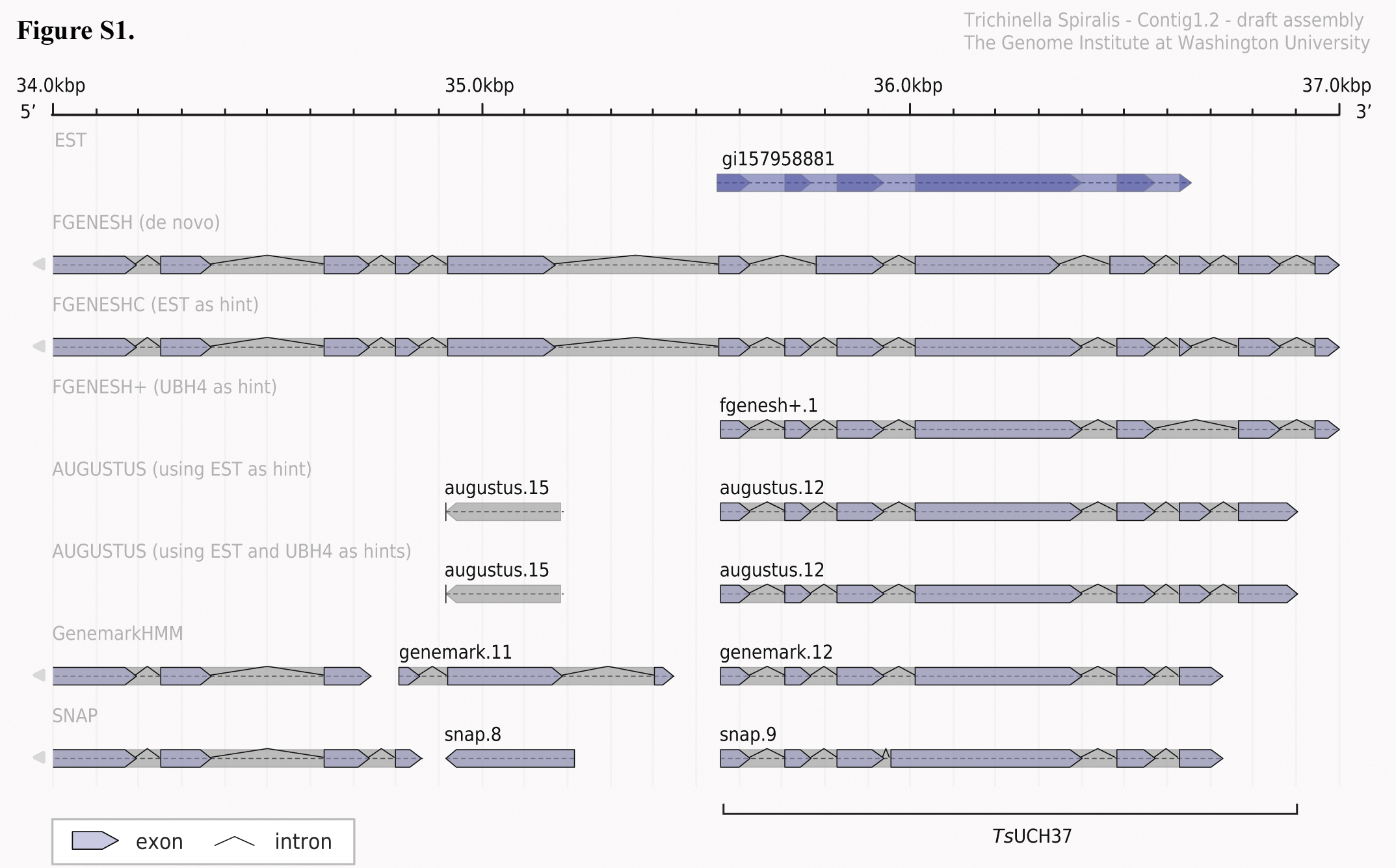

Supplement: Figure S1 — Gene predictions of the putative TsUCH37 ORF. The EST fragment obtained by LC/MS/MS (gi157958881) was aligned with contig 1.2 from the draft assembly of the T. spiralis genome. At this location, gene predictions from Fgenesh (de novo or using EST as an hint/constraint as Fgenesh+) span a large region of the contig. However, when employing UBH4 (the C. elegans UCH-L5 orthologue) as a hint, the Fgenesh_C predicted start agrees with all other gene prediction programs employed (AUGUSTUS de novo, AUGUSTUS with EST and/or UBH-4 as constraints, SNAP, Genemark.HMM). (TIF) [file pntd.0001340.s001.tif]

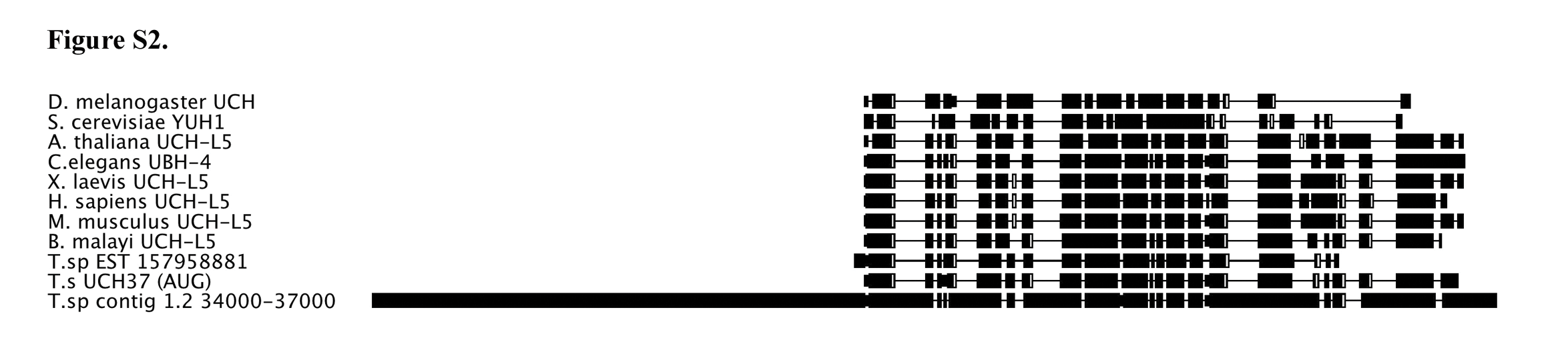

Supplement: Figure S2 — Multiple nucleotide alignments (MUSCLE) of putative UCH-L5 orthologues with the T. spiralis contig 1.2 (34000-37000), the AUGUSTUS predicted ORF for TsUCH37 and the EST gi157958881. The AUGUSTUS TsUCH37 sequence, EST gi157958881 and the orthologous UCH-L5 sequences are coding sequence only (no introns). The Contig was obtained from the Genome Institute at Washington University, accessed August 2010. The alignment was generated using Geneious (Drummond AJ et al, www.geneious.com). Blocks indicate nucleotide bases and lines indicate gaps in alignment. Accession numbers for coding sequences in order of appearance: D.m NM_001201752, S.c NM_001181757, A.t NM_105238, C.e NM_063283, X.l NM_001095597, H.s NM_001199263, M.m NM_019562, B.m XM_001895545. (TIF) [file pntd.0001340.s002.tif]
